# Will Preoperative Atrophy and Fatty Degeneration of the Shoulder Muscles Improve after Rotator Cuff Repair in Patients with Massive Rotator Cuff Tears?

**DOI:** 10.1155/2012/195876

**Published:** 2012-01-12

**Authors:** Hiroshi Yamaguchi, Naoki Suenaga, Naomi Oizumi, Yoshihiro Hosokawa, Fuminori Kanaya

**Affiliations:** ^1^Department of Orthopedic Surgery, Graduate School of Medicine, University of the Ryukyus, 207 Uehara, Nishihara, Okinawa 903-0215, Japan; ^2^The Upper Extremity Center of Joint Replacement and Endoscopic Surgery, Hokushin Orthopaedic Hospital, Sapporo, Hokkaido 003-0823, Japan; ^3^Department of Orthopaedic Surgery, Kaisei Hospital, Obihiro, Hokkaido 080-2473, Japan

## Abstract

Recently, retear rate after repair for massive cuff tear have been improved through devised suture techniques. However, reported retear rate is relevant to preoperative atrophy and fatty degeneration. The purpose of this study was to investigate whether preoperative atrophy and fatty degeneration of rotator cuff muscles improve by successful repair. Twenty-four patients with massive rotator cuff tear were evaluated on the recovery of atrophy and fatty degeneration of supraspinatus and infraspinatus muscle after surgery. Atrophy was classified by the occupation ratio and fatty degeneration by modified Goutallier's classification. Both were assessed on magnetic resonance imaging (MRI) before and after the operation. When the cuff was well repaired, improvement of the atrophy and fatty degeneration were observed in a half and a one-fourth of the cases, respectively. In retear cases, however, atrophy and fatty degeneration became worse. Improvement of atrophy and fatty degeneration of the rotator cuff muscles may be expected in the cases with successful achievement of rotator cuff repair for large and massive tear.

## 1. Introduction

Arthroscopic and open repair of the rotator cuff yield variable healing rates [1–6]. Large and massive tears are known to have less satisfactory results, because chronic large and massive tears often involve atrophy and fatty degeneration of the muscles [2–6]. On the other hand, recent studies with devised suture techniques report higher healing rates [1, 7]. However, it has been yet unclear whether successful cuff repair shows recovery of atrophy and fatty degeneration of cuff muscles.

The purpose of this study was to investigate postoperative improvements of atrophy and fatty degeneration of the cuff muscles and their relationships with cuff repair integrity.

## 2. Materials and Methods

We treated 29 shoulders of 29 patients with chronic massive rotator cuff tears by the surface-holding repair technique with transosseous suture [7] between 2001 and 2007. The criteria for operative repair included (1) at least six months of failed nonoperative treatment, except for the actual trauma, with the patient continuing to complain of subjectively unacceptable pain or disability, or both, (2) patient need/desire to use the arm at or above the level of the head, (3) good motivation to comply with the postoperative treatment regimen, and (4) the absence of moderate-to-marked osteoarthritis (OA). Twenty-four patients and/or their families agreed to undergo follow-up investigations for more than 18 months after the surgery. The follow-up rate was 82.8% (24 of 29 shoulders). There were 17 men and 7 women, with a mean age at the time of surgery of 63.4 years (range, 45–82 years). The preoperative tear size was assessed by Cofield's classification at the time of the surgery [8]. The torn tendons were of the supraspinatus and infraspinatus in 18 shoulders, the supraspinatus, infraspinatus, and partial subscapularis in 5 shoulders, and the supraspinatus, infraspinatus, and a part of teres minor in 1 shoulder. The average follow-up period was 38.9 months (range, 18–71 months).

### 2.1. Operative Technique and Rehabilitation Protocol

**Figure 1:**
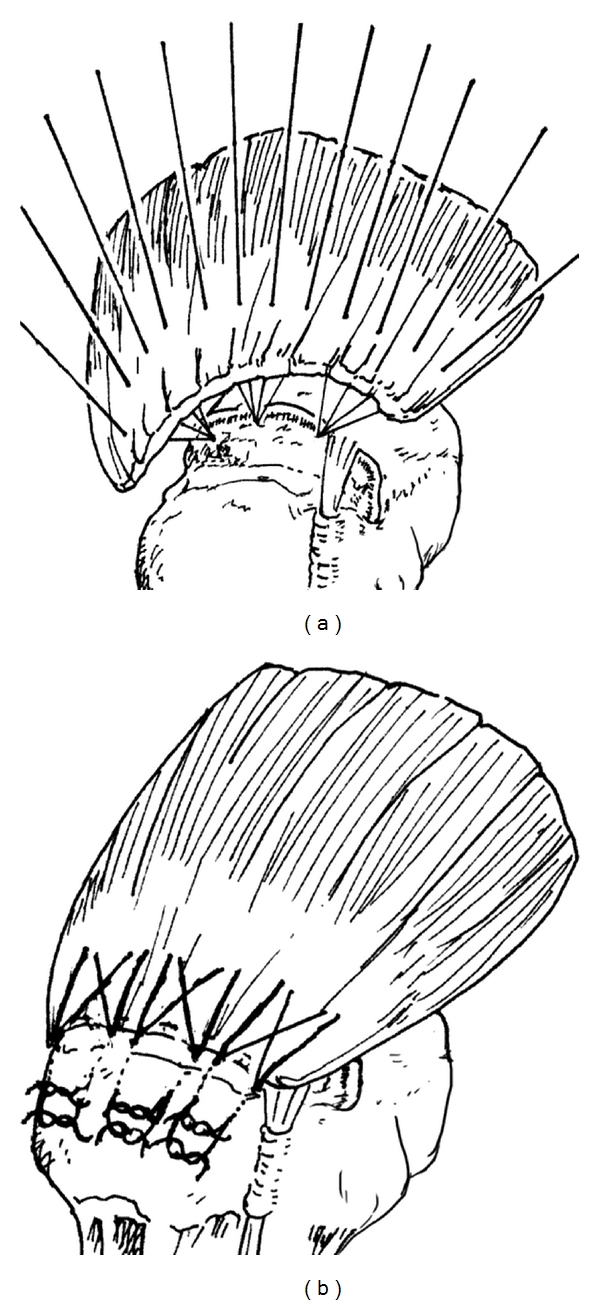
Suture anchors were placed on the medial site of the “footprint” (a) A bone trough was made approximately 1 cm proximal to the greater tuberosity until the cancellous bone was exposed. Threads from the anchors were pulled out to the lateral cortex and tied without tying on the tendon (b).

After the skin incision, the deltoid was split between the anterior and middle fibres, and a portion of the anterior fibres was detached from the acromion. Acromionplasty and resection of coracoacromial ligament were performed. Extra-articular and intra-articular soft-tissue release was performed to obtain sufficient mobility of the tendon. If tendons were too retracted to be reattached to the greater tuberosity with the arm positioned at the side, medial attachment, that is, at approximately 10 millimeters, was employed. If the tendon did not reach the medial advanced area of the humeral head, we have performed latissimus dorsi muscle transfer [9]. Because medial advancement of 17 millimeters or more may reduce the “moment arm” of the shoulder [10, 11]. A bone trough was made about 1 cm proximal to the greater tuberosity until the cancellous bone was exposed. Metal suture anchors were placed on the proximal site of the “footprint” to enlarge the contact area of the tendon on the bony surface, and the distance to the anchor in all cases was approximately 1.5 cm (Figure 1(a)). Two threads from each anchor were pulled out to the lateral cortex and tied without tying on the tendon (Figure 1(b)).

 An abduction pillow was used for eight weeks postoperatively. A systematic postoperative rehabilitation program was carried out using passive range of motion (ROM) exercises starting two weeks after surgery. Active elevation in a sitting position from the adducted position of the shoulder was permitted starting at 10 weeks. Isometric cuff exercises were allowed starting at 12 weeks. Heavy work or sports were permitted after six months postoperatively, after assessing the recovery of muscle strength and ROM.

### 2.2. Evaluation

Evaluation of the recovery of atrophy and fatty degeneration of the rotator cuff muscles (supraspinatus and infraspinatus) was performed for 24 shoulders by magnetic resonance imaging (MRI) obtained before the surgery and at the final followup. Two orthopedic surgeon, except for chief operator, analyzed the evaluations. For analyzing atrophy, we examined oblique sagittal views of T2-weighted MRI obtained about 20 mm proximal to the deepest point on the concave curve of the glenoid surface (Figure 1), as modified from Shen et al.'s report [12]. Using the Image J image analyzing software (National Institutes of Health, Baltimore, Md, USA), we measured the actual occupied area and the estimated anatomical area of the supraspinatus and infraspinatus muscles (2 shoulders in which we could not differentiate between the infraspinatus and teres minor were excluded). We calculated the occupation ratio (= actual occupied area/estimated anatomical area × 100) [13] and compared the results before and after surgery. According to the occupation ratio, we classified the severity of atrophy into 4 grades: Grade 1, 75% or more; Grade 2, 50%–74%; Grade 3, 25%–49%; Grade 4, 24% or less.

**Figure 2:**
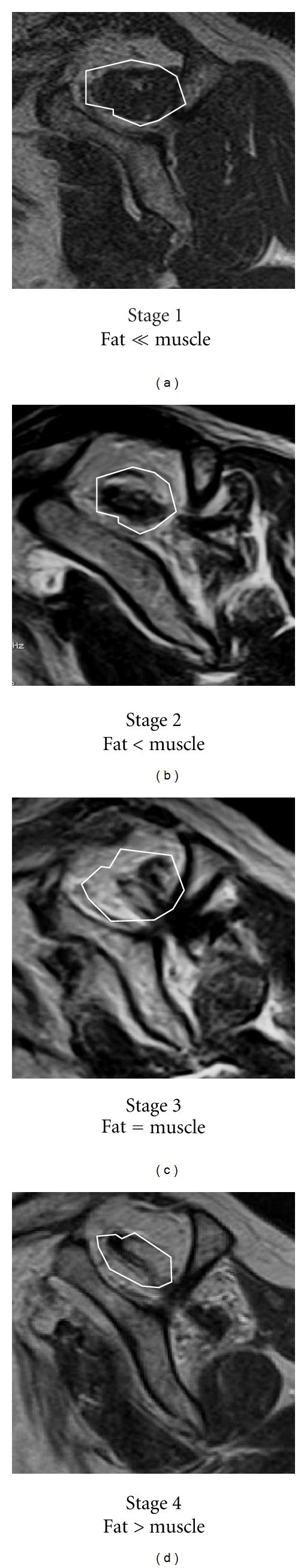
Modified Goutallier's classification. Examples show each stage of fatty degeneration in supraspinatus, which enclosed white line.

**Figure 3:**
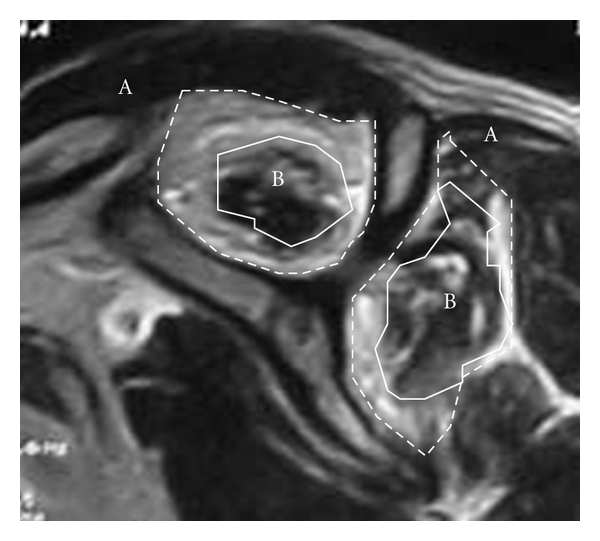
Occupation ratio = actual occupied area (B)/estimated anatomical area (A) ×100.

For investigating fatty degeneration, we used modified Goutallier's classification [14] to classify the “actual occupied area” into 5 stages: stage 0, completely normal muscle without any fatty streaks; stage 1, some fatty streaks in the muscle; stage 2, pronounced fatty infiltration, but muscle area still exceeding the fat area; stage 3, fat area equal to muscle area; stage 4, fat area exceeding the muscle area (Figures 2 and 3).

The repair integrity was classified into 5 types on oblique coronal and oblique sagittal views of T2-weighted MRI, on the basis of Sugaya's system [5]: type 1, repaired cuff of sufficient thickness with a homogeneously low intensity in each image; type 2, sufficient thickness associated with a partial high-intensity area; type 3, insufficient thickness without discontinuity; type 4, minor discontinuity in more than one slice, suggestive of a small tear; type 5, major discontinuity in each image, suggestive of a medium-to-large tear.

All patients were assessed using the scoring system of the Japanese Orthopaedic Association (JOA score). The JOA score is a 100-point scoring system, with 30 points for pain, 20 for function, 30 for ROM, and 20 for radiographic findings and stability. The JOA scores were evaluated preoperatively and at the final followup.

Statistical analysis was performed using the Wilcoxon's test, with *P* < 0.05 considered to indicate statistical significance. 

## 3. Results

### 3.1. Atrophy

The average preoperative and postoperative occupation ratios were 36.1% (range, 17.5%–58.9%) and 49.4% (range, 16.6%–74.0%) in the supraspinatus, and 56.8% (range, 20.2%–92.2%) and 65.3% (range, 7.4%–90.5%) in the infraspinatus, respectively, being statistically significant in both (supraspinatus, *P* = 0.0003; infraspinatus, *P* = 0.0459). Preoperative atrophy of the cuff muscles was observed in all shoulder, in supraspinatus: grade 2, 4 shoulders; grade 3, 16 shoulders; grade 4, 4 shoulders, and ininfraspinatus: grade 1, 3 shoulders; grade 2, 13 shoulders; grade 3, 5 shoulders; grade 4, 1 shoulder (Figure 6).

Grade of atrophy at followup was supraspinatus, grade 1, 1 shoulder; grade 2, 12 shoulders; grade 3, 9 shoulders; grade 4, 2 shoulders, and in infraspinatus: grade 1, 8 shoulders; grade 2, 10 shoulders; grade 3, 3 shoulders; grade 4, 1 shoulder (Table 1).

### 3.2. Fatty Degeneration

Preoperative fatty degeneration of the cuff muscles was observed in all shoulders, in supraspinatus: stage 1, 8 shoulders; stage 2, 6 shoulders; stage 3, 10 shoulders, and in infraspinatus: stage 1, 7 shoulders; stage 2, 2 shoulders; stage 3, 13 shoulders (Figure 6)

Severity of fatty degeneration at followup, in supraspinatus: stage 1, 8 shoulders; stage 2, 10 shoulders; stage 3, 4 shoulders; stage 4, 2 shoulders, and in infraspinatus: stage 1, 4 shoulders; stage 2, 11 shoulders; stage 3, 5 shoulders; stage 4, 2 shoulders (Figure 6).

### 3.3. Repair Integrity

Repair integrity was categorized on the basis of the MRI findings: type 1, 13 shoulders (54.2%); type 2, 3 shoulders (12.5%); type 3, 5 shoulders (20.8%); type 4, 3 shoulders (12.5%); type 5, none (0%). Because types 4 and 5 are regarded as retear, the 3 shoulders (12.5%) with type 4 were diagnosed as retears in this study. The overall successful repair rate was 87.5%.

### 3.4. Clinical Outcome

The JOA score reflected significant improvement of the status of the shoulders at the final followup after the surgery (*P* < 0.05). The average total JOA score increased from 53.3 points (range, 40–67 points) preoperatively to 90.9 points (range, 77–100) postoperatively.

### 3.5. Comparison of Preoperative/Postoperative Atrophy and Fatty Degeneration

#### 3.5.1. Supraspinatus

Atrophy improved in 11 shoulders and fatty degeneration in 6; in particular, atrophy improved by 2 grades in 3 of 11 shoulders. However, 2 shoulders showed progressive atrophy, and 3 showed progressive fatty degeneration (Figure 6).

#### 3.5.2. Infraspinatus

Atrophy improved in 10 shoulders and fatty degeneration in 6. However, 3 shoulders showed progressive atrophy, and 5 showed progressive fatty degeneration (Figure 6).

2 shoulders of 3 retear cases had progression of both atrophy and fatty degeneration.

### 3.6. Relationship between Preoperative Atrophy and Repair Integrity

#### 3.6.1. Supraspinatus

Of 4 shoulders with preoperative grade 2 atrophy, the repair integrity was type 1 in 3 shoulders and type 2 in 1. Of 16 shoulders with grade 3 atrophy, it was type 1, type 2, type 3, and type 4 in 8, 2, 3, and 3 shoulders, respectively. Of 4 shoulders with grade 4 atrophy, it was type 1 in 2 shoulders and type 3 in 2 (Table 1).

#### 3.6.2. Infraspinatus

Of 3 shoulders with preoperative grade 1 atrophy, the repair integrity was type 1 in 2 shoulders and type 2 in 1. Of 13 shoulders with grade 2 atrophy, it was type 1, type 2, type 3, and type 4 in 8, 0, 3, and 2 shoulders, respectively. Of 5 shoulders with grade 3 atrophy, it was type 1 in 3 shoulders and type 3 in 2. In the one shoulder with grade 4 muscle atrophy, the repair integrity was type 4 (Table 1). 

In both muscles, there were no significant correlation between atrophy and repair integrity.

### 3.7. Relationship between Preoperative Fatty Degeneration and Repair Integrity

#### 3.7.1. Supraspinatus

Of 8 shoulders with preoperative stage 1 fatty degeneration, the repair integrity was type 1 in 3 shoulders, type 2 in 3, type 3 in 1, and type 4 in 1. Of 6 shoulders with stage 2 fatty degeneration, it was type 1 in 3 shoulders, type 2 in 2, and type 4 in 1. Of 10 shoulders with stage 3 fatty degeneration, it was type 1 in 7 shoulders, type 3 in 2, and type 4 for in 1 (Table 1).

#### 3.7.2. Infraspinatus

Of 7 shoulders showing preoperative stage 1 fatty degeneration, the repair integrity was type 1 in 2 shoulders, type 2 in 3, type 3 in 1, and type 4 in 1. Of 2 shoulders showing stage 2 fatty degeneration, repair integrity was type 1 in 1, type 3 in 1. Of 13 shoulders showing stage 3 fatty degeneration, it was type 1 in 8 shoulders, type 3 in 3, and type 4 in 2 (Table 1). 

In both muscles, there were no significant correlation between fatty degeneration and repair integrity.

### 3.8. Complications

There were no intraoperative or perioperative complications, such as neural injury, wound infection, or suture anchor problems.

## 4. Case Presentations

**Case 1**

**Figure 4:**
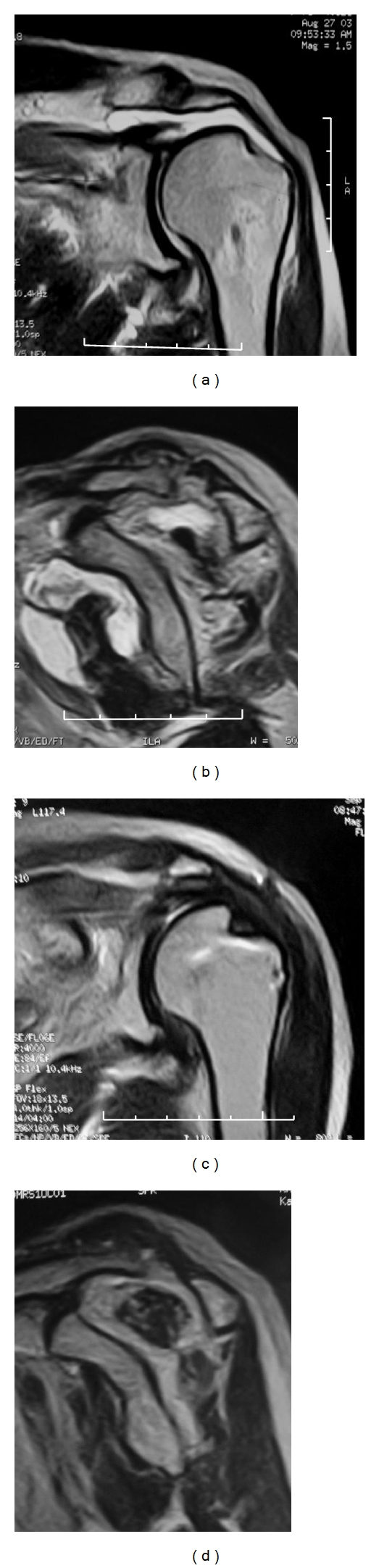
Case 1. (a) Pre-op MRI; oblique coronal, (b) oblique sagittal. (c) Post-op MRI (48 months after surgery); oblique coronal, (d) oblique sagittal.

A 72-year-old woman with supraspinatus and infraspinatus tendon tears. MRI was performed preoperatively and 48 months after repair surgery. The JOA score improved from 61 to 94. The repair integrity was type 1 according to Sugaya's classification. The preoperative and postoperative occupation ratios were 17.3% (grade 4) and 49.5% (grade 3) for the supraspinatus, and 35.3% (grade 3) and 54.8% (grade 2) for the infraspinatus, respectively. Fatty degeneration improved from stage 3 to stage 2 in the supraspinatus, and from stage 3 to stage 2 in the infraspinatus (Figure 4).

**Case 2**

**Figure 5:**
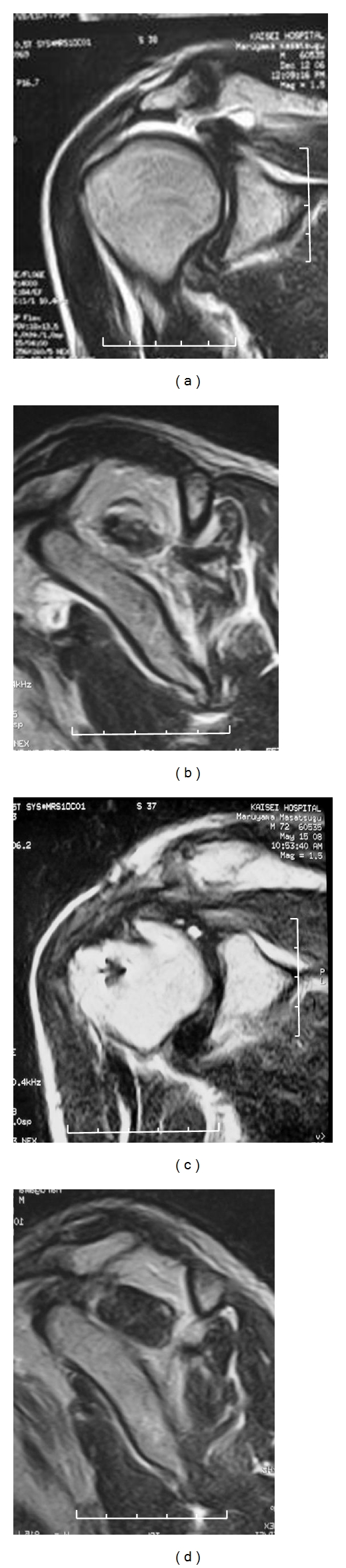
Case 2. (a) Pre-op MRI; coronal, (b) sagittal. (c) Post-op MRI (18 months after surgery); coronal, (d) sagittal.

**Figure 6:**
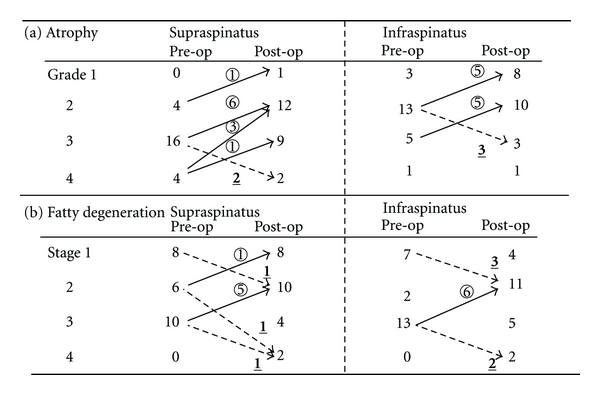
Arrows and numbers within the circle mean improvement, dotted arrows and numbers with underbur mean aggravation.

A 71-year-old man with supraspinatus, infraspinatus, and partial subscapularis tendon tears. MRI was performed preoperatively and 18 months after the surgery. The JOA score improved from 41 to 93. The repair integrity was type 1. The preoperative and postoperative occupation ratios were 24.4% (grade 4) and 52.1% (grade 2) for the supraspinatus, and 46.4% (grade 3) and 63.5% (grade 2) for the infraspinatus. Fatty degeneration improved from stage 3 to stage 2 in the supraspinatus, and from stage 3 to stage 2 in the infraspinatus (Figure 5).

## 5. Discussion

Previous studies reported the following important factors determining the repair integrity after rotator cuff repair: tear size [15], location, presence/absence of atrophy and fatty degeneration in the muscles [16, 17], repair tension, tendon quality, and patient age [2]. Gerber et al. [16, 18] and Goutallier et al. [14, 16, 17] reported that the most significant risk factors for retear are the presence of atrophy and fatty degeneration.

Recent biomechanical studies have demonstrated that the elements for successful repair of a rotator cuff tear are achievement of strong fixation [19–21], a high interface pressure, a wide interface area between the tendon and the bone [22, 23], and minimization of stress concentration inside the tendon [7, 24]. Some new suture techniques allowing achievement of all of these elements have been devised [1, 7]. Therefore, the reported retear rate after open and arthroscopic repair surgeries has improved [1, 25]. Then, improvement of rotator cuff repair techniques is also expected to facilitate recovery of the muscle atrophy and fatty degeneration. 

However, in some basic studies, Matsumoto et al. found neither reversal of atrophy nor reversal of fatty infiltration after delayed repair in rabbits [26]. Burkhead et al. reported that successful repair may partially reverse muscular atrophy but not fatty infiltration in sheep [19]. In a previous clinical study, Gerber et al. reexamined the records of 57 of 63 patients who underwent postoperative CT and were followed up for a mean duration of 17.7 months [18]. They found no regression of infraspinatus fatty degeneration even after a watertight repair, and improvement of supraspinatus fatty degeneration was noted in only 6 cases [14]. Thomazeau et al. reported more optimistic results of evaluation of supraspinatus muscle atrophy: in one half of the 22 patients who underwent continuous cuff repair, the atrophy improved by more than 10% [27] (mean followup, 21.1 months).

In this study, we found higher rate of improvement of atrophy and no significant correlation between the grade of preoperative atrophy and the repair integrity. And we also found higher rate of improvement of fatty degeneration and no significant correlation between preoperative fatty degeneration and repair integrity. We consider that the reasons for this result are due to our higher rate of successful repair and longer follow-up period, giving enough time for atrophy and fatty degeneration to improve.

Several limitations must be considered when changes in the rotator cuff muscles are analyzed by determining crossed-sectional areas on one MRI plane. One crossed-sectional area may not represent the total muscle volume, especially as muscles change in shape along their length. Furthermore, we did not investigate time-dependent changes in this study, and there is some possibility that the measured area was not exactly same before and after surgery because of the influence of retraction of the cuff muscles on MRI [28, 29]. In the future study, sequential postoperative MRI must be performed to investigate under what circumstances the fatty degeneration might be irreversible and clinical point at which cuff muscles may not be able to return to nearly normal function despite successful surgical repair as evaluated by MRI.

In conclusion, we indicate that successful repair of chronic massive cuff tears may allow arrest or recovery of severe fatty degeneration and atrophy of the torn muscles. Furthermore, in massive cuff tears, successful repair is the key to long-term functional improvement not only for pain relief and stabilizing.

## Figures and Tables

**Table 1:** Relationship between preoperative atrophy (a)/fatty degeneration (b) and the repair integrity.

		(a) Atrophy				(b) Fatty degeneration			
		Ocupation ratio				Goutallier's classification			
		Grade 1	Grade 2	Grade 3	Grade 4	Stage 1	Stage 2	Stage 3	Stage 4
	Type	SSP/ISP	SSP/ISP	SSP/ISP	SSP/ISP	SSP/ISP	SSP/ISP	SSP/ISP	SSP/ISP
Sugaya's classification	I	0./2	3./8	8./3	2./0	3./2	3./1	7./8	
	II	0./1	1./0	2./0		3./3			
	III		0./3	3./2	2./0	1./1	2./1	2./3	
	IV		0./2	3./0	0./1	1./1	1./0	1./2	
	V								
